# Supplementary material for: scFFPE-ATAC enables high-throughput single cell chromatin accessibility profiling in formalin-fixed paraffin-embedded samples
Source: Nat Commun. 2025 Nov 14;16:10022. doi: 10.1038/s41467-025-66170-4 (PMC12618699; doi:10.1038/s41467-025-66170-4)
Supplement: Supplementary file 1 — Supplementary Information [file 41467_2025_66170_MOESM1_ESM.pdf]

Supplementary information:

scFFPE-ATAC enables high-throughput single cell chromatin accessibility profiling in formalin-fixed paraffin-embedded samples

Ram Prakash Yadav[1,#], Pengwei Xing[1,#], Miao Zhao[1], Peter Hollander[1], Carina Strell[1,2], Minglu Xie[1], Maede Salehi[1], Emma Torell[1], Tobias Sjöblom[1], Gunilla Enblad[1], Rose-Marie Amini[1], Fredrik Johansson Swartling[1], Ingrid Glimelius[1], Patrick Micke[1], Mats Hellström[1], Xingqi Chen[1],*

1, Department of Immunology, Genetics and Pathology, Uppsala University, Uppsala, Sweden
2, Centre of Cancer Biomarkers (CCBIO), Department of Clinical Medicine, University of Bergen, Bergen, Norway
# Contributed equally to this work
*For correspondence: xingqi.chen@igp.uu.se, Phone: +46-184714072

Supplementary Figure 1-21.

Supplementary Technical Note 1-2.

Supplementary Table 1-24.

Supplementary Figures 1-21:

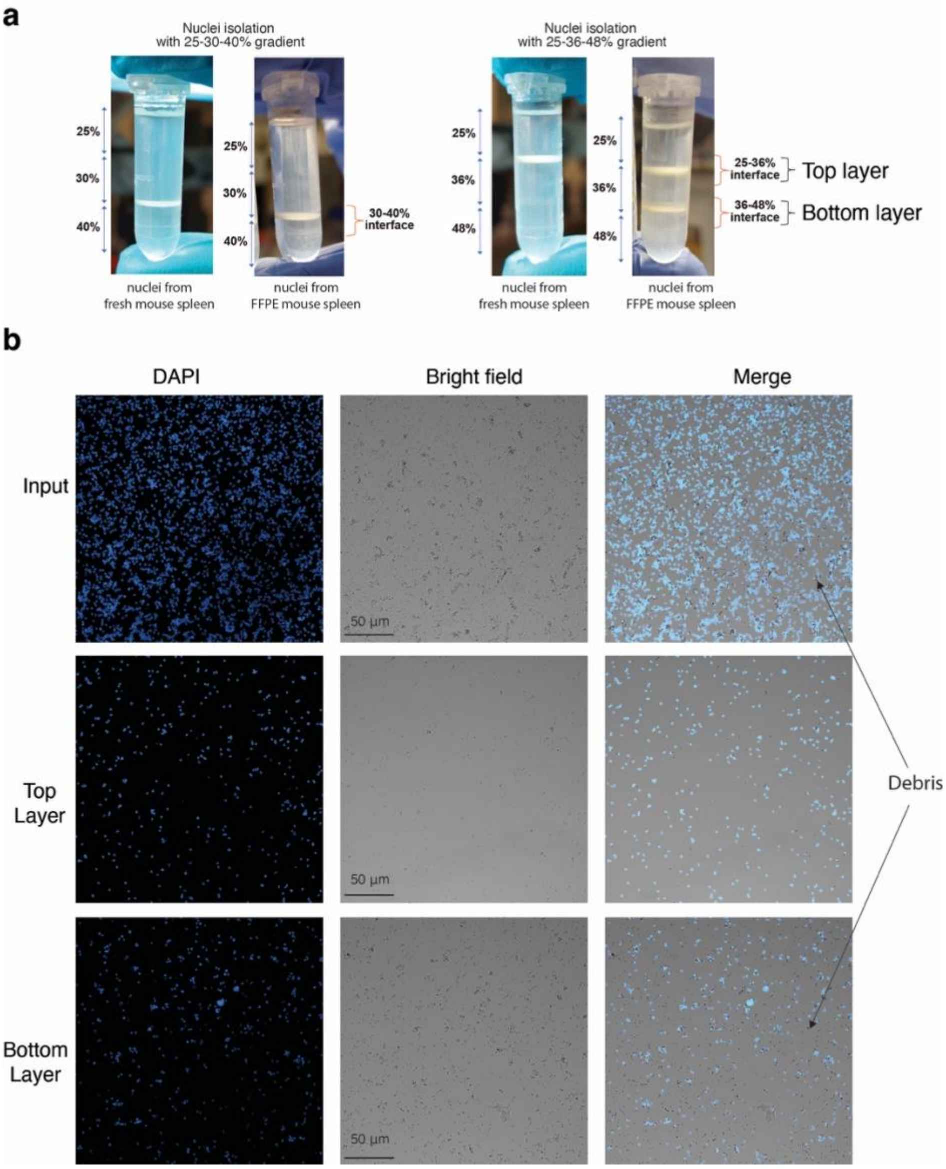

Supplementary Figure 1: **Optimization of nuclei isolation from mouse spleen FFPE samples**

a. Density gradient centrifugation of mouse spleen FFPE nuclei under two different density gradient layer conditions, comparing fresh and FFPE samples. Left panel: Condition 1; Right panel: Condition 2.

b. Representative microscopy images of purified mouse spleen FFPE nuclei from the input, top layer, and bottom layer of Condition 2 shown in panel **a**. Three independent experiments were performed, showing consistent results.

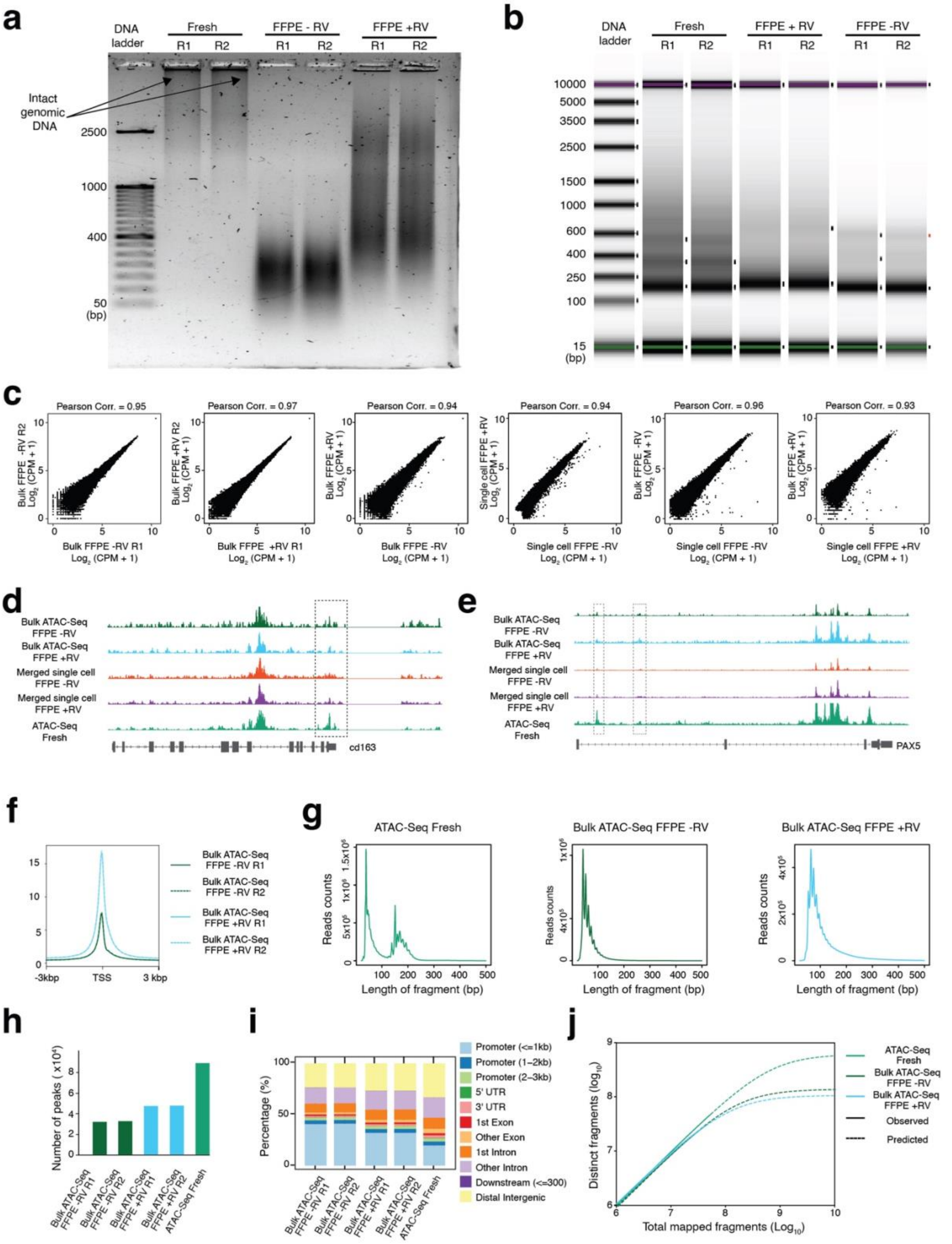

Supplementary Figure 2: Conventional ATAC-seq and scATAC-seq analysis of FFPE mouse spleen samples

a. Length distribution of purified genomic DNA from fresh mouse spleen nuclei, FFPE mouse spleen nuclei without reverse crosslinking (−RV), and FFPE mouse spleen nuclei with reverse crosslinking (+RV). Each condition includes two technical replicates (R1 and R2). The entire gel is shown without cropping. Three independent experiments were performed, showing consistent results.

b. Length distribution of PCR amplicons from conventional ATAC-seq performed on fresh mouse spleen nuclei, FFPE mouse spleen nuclei without reverse crosslinking (−RV), and FFPE mouse spleen nuclei with reverse crosslinking (+RV). Each condition includes two technical replicates (R1 and R2). Three independent experiments were performed, showing consistent results.

c. Genome-wide correlation of sequencing reads within accessible chromatin peaks between conventional ATAC-seq and scATAC-seq across fresh mouse spleen nuclei, FFPE mouse spleen nuclei without reverse crosslinking (−RV), and FFPE mouse spleen nuclei with reverse crosslinking (+RV).

d. Genome browser tracks of the myeloid cell marker gene *Cd163* from conventional ATAC-seq and scATAC-seq across experimental conditions. Dotted squares highlight peaks present in fresh samples but absent in FFPE samples.

e. Genome Genome browser tracks of the B cell marker gene *Pax5* from conventional ATAC-seq and scATAC-seq across experimental conditions. Dotted squares indicate peaks detected in fresh samples but absent in FFPE samples.

f. Transcription start site (TSS) enrichment scores from conventional ATAC-seq across different experimental conditions.

g. Fragment length distribution of sequencing reads from conventional ATAC-seq across experimental conditions.

h. Number of peaks identified by conventional ATAC-seq in FFPE and fresh mouse spleen nuclei. Each bar represents one sample, and technical replicates are labeled. Each condition includes two technical replicates (R1 and R2).

i. Genomic annotation of ATAC-seq peaks identified by conventional ATAC-seq in FFPE and fresh mouse spleen nuclei.

j. Library complexity estimation of conventional ATAC-seq in FFPE and fresh mouse spleen nuclei.

Source data are provided as a Source Data file for Supplementary Fig. 2c-j.

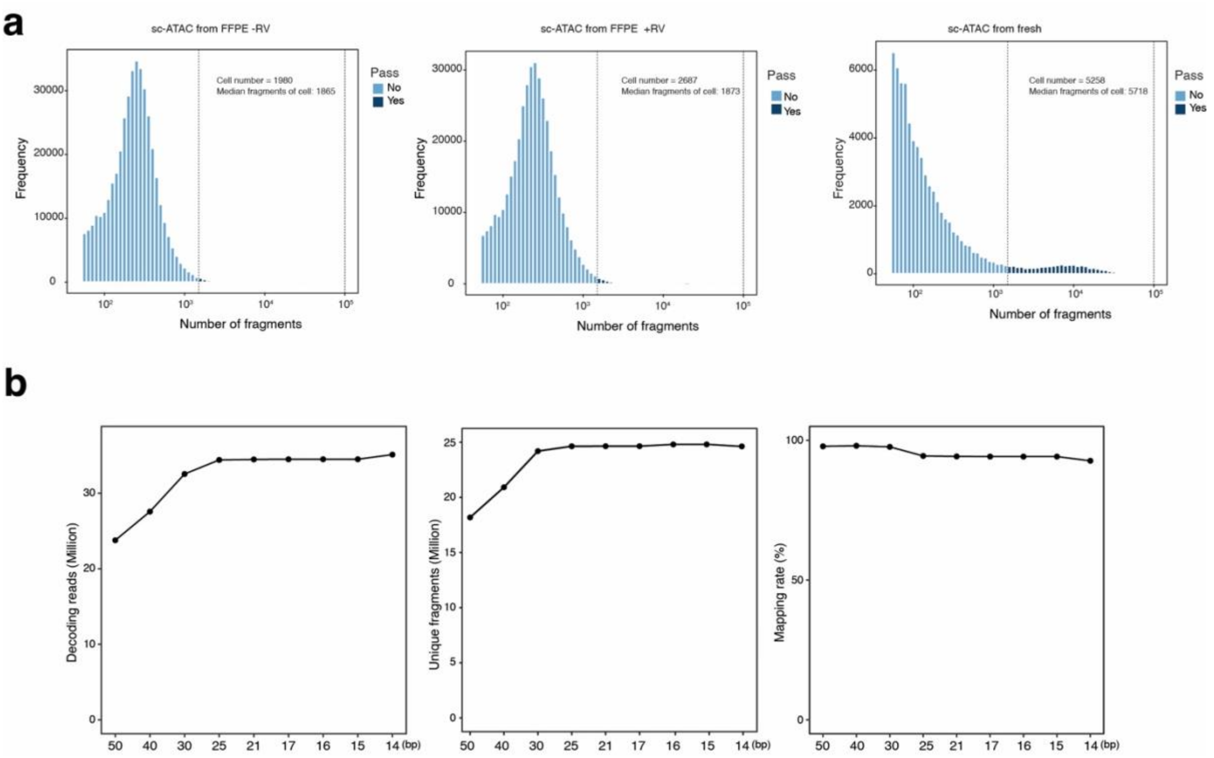

Supplementary Figure 3: Comparative analysis of conventional scATAC-seq between FFPE and fresh mouse spleen nuclei.

a. Frequency distribution of fragment counts from mouse FFPE spleen without reverse crosslinking (−RV) (left panel), mouse FFPE spleen with reverse crosslinking (+RV) (middle panel), and published fresh mouse spleen data (right panel).

b. Optimization of fragment length for mapping using 50 million fragments from scATAC-seq libraries of mouse FFPE spleen samples. Left panel: number of decoded fragments; middle panel: number of unique fragments; right panel: mapping rate.

Source data are provided as a Source Data file for Supplementary Fig. 3a, 3b.

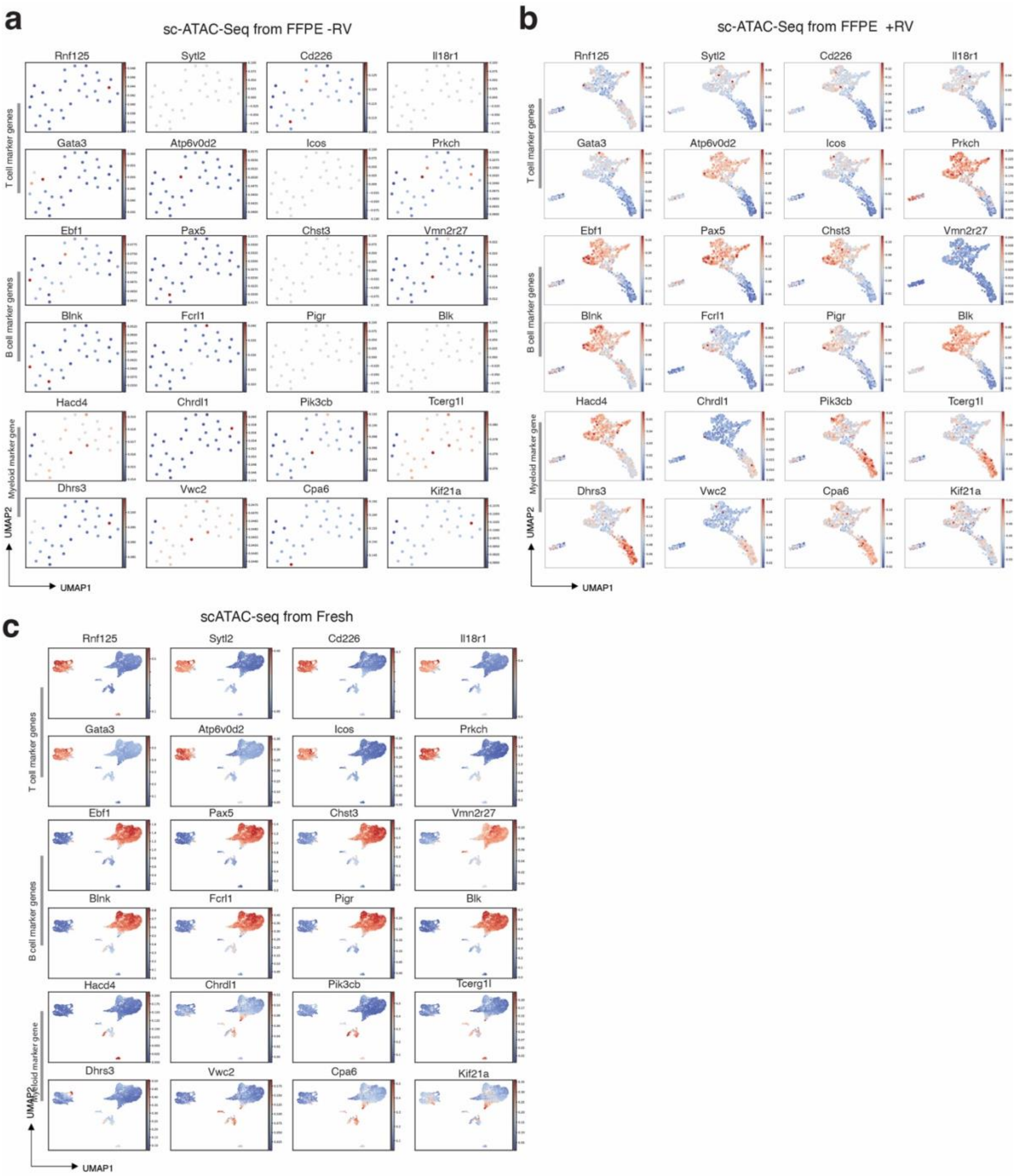

Supplementary Figure 4: Gene activity profiles from conventional scATAC-seq in different conditions: **(a)** FFPE mouse spleen without reverse crosslinking (−RV); **(b)** FFPE mouse spleen with reverse crosslinking (+RV), and **(c)** fresh mouse spleen nuclei.
Source data are provided as a Source Data file for Supplementary Fig. 4a-c.

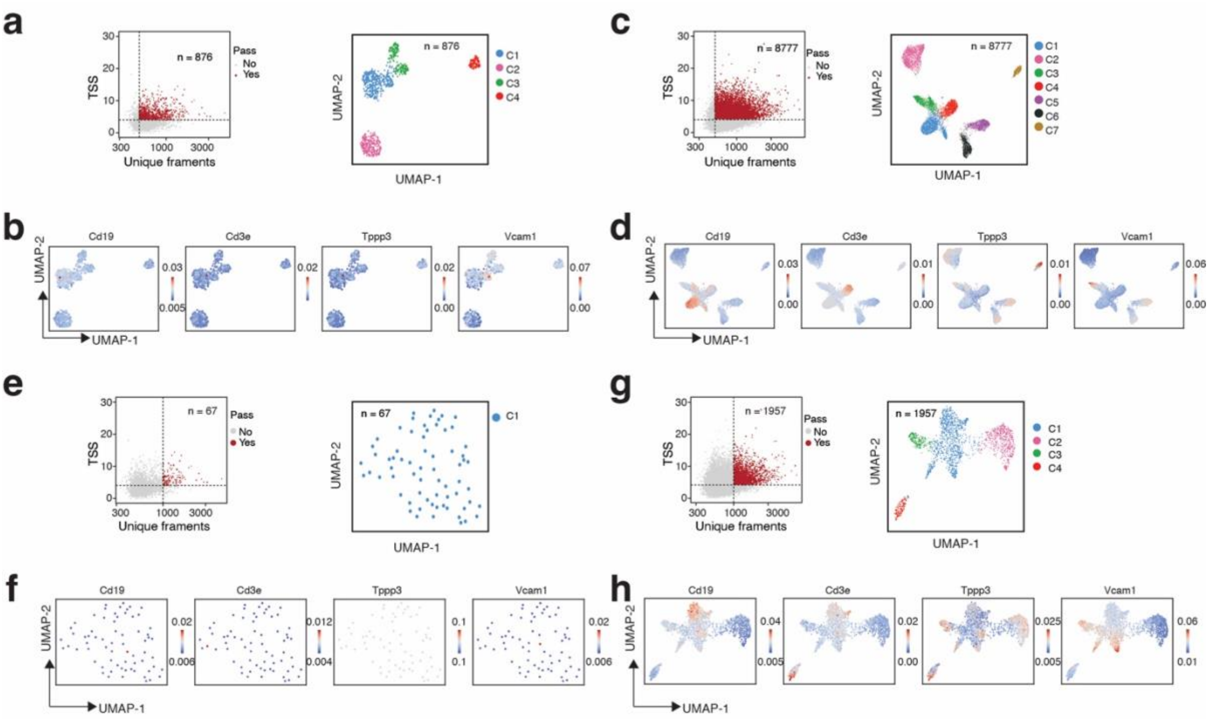

Supplementary Figure 5: Conventional scATAC-seq applied to FFPE samples with reduced parameter cutoffs

a. Left: QC-passing cells (≥ 500 frags/cell, TSS ≥ 4, −RV); Right: Cell clusters (UMAP).
b. Cell-type marker gene (*Cd19*/*Cd3e*/*Tppp3*/*Vcam1*) activity in -RV clusters (≥500 frags/cell).
c. Left: QC-passing cells (≥ 500 frags/cell, TSS ≥ 4, +RV); Right: Cell clusters (UMAP).
d. Cell-type marker gene (*Cd19*/*Cd3e*/*Tppp3*/*Vcam1*) activity in +RV clusters (≥500 frags/cell).
e. Left: QC-passing cells (≥ 1000 frags/cell, TSS ≥ 4, −RV); Right: Cell clusters (UMAP).
f. Cell-type marker gene (*Cd19*/*Cd3e*/*Tppp3*/*Vcam1*) activity in -RV cell clusters (≥1000 frags/cell).
g. Left: QC-passing cells (≥ 1000 frags/cell, TSS ≥ 4, +RV); Right: Cell clusters (UMAP).
h. Cell-type marker gene (*Cd19*/*Cd3e*/*Tppp3*/*Vcam1*) activity in +RV cell clusters (≥1000 frags/cell).

Source data are provided as a Source Data file for Supplementary Fig. 5a-h.

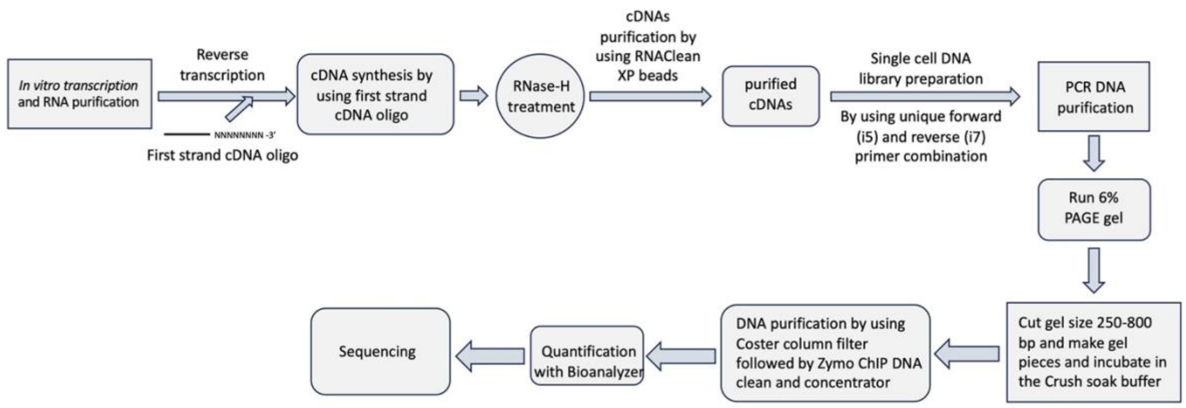

Supplementary Figure 6: The workflow of scFFPE-ATAC sequencing library construction.

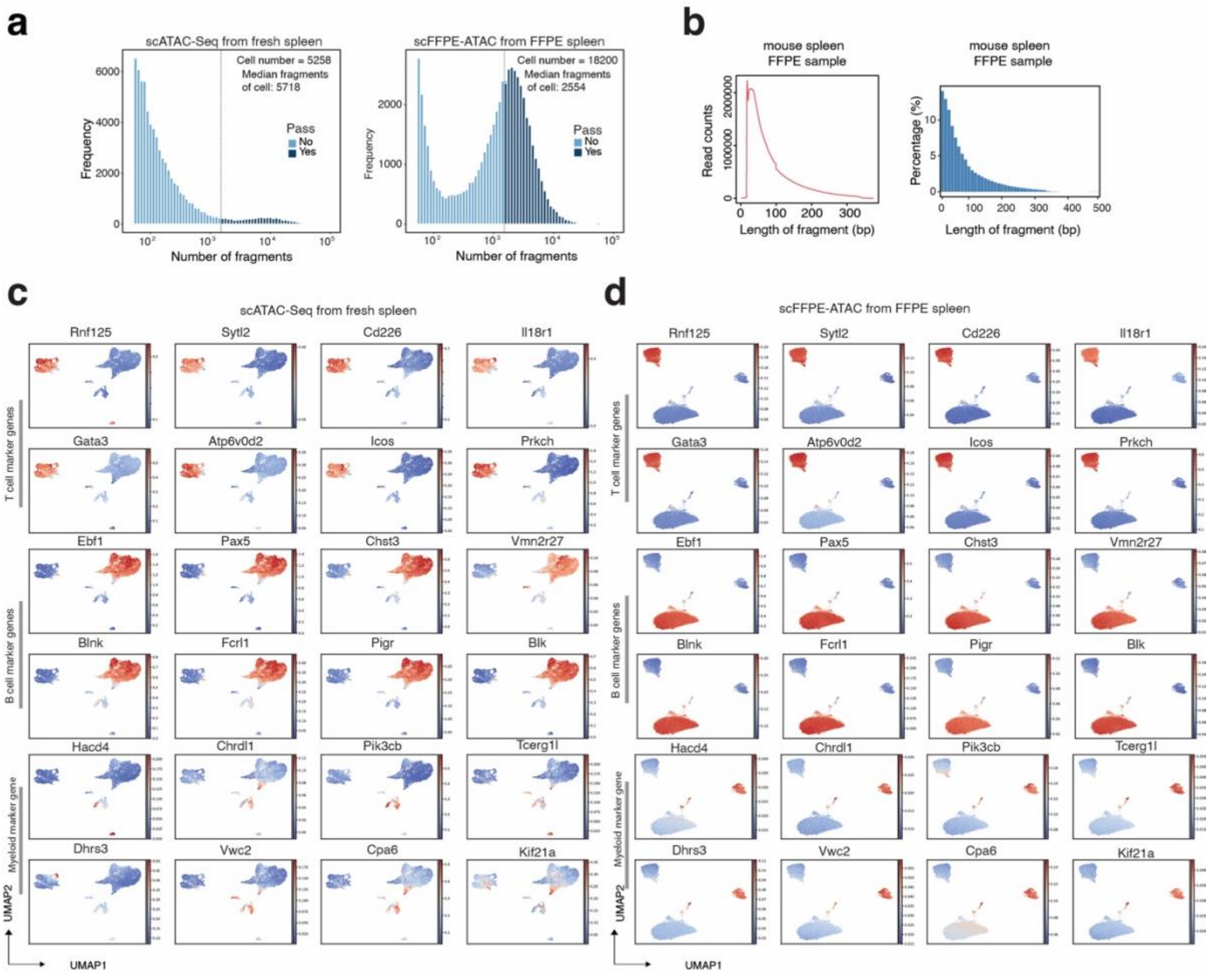

Supplementary Figure 7: **Comparison of single-cell chromatin accessibility between fresh using conventional scATAC-Seq and FFPE-preserved mouse spleen with scFFPE-ATAC**.

a. Fragment count frequency distributions from: (Left) fresh mouse spleen (conventional scATAC-Seq) and (Right) FFPE mouse spleen (scFFPE-ATAC).

b. Fragment size distribution of scFFPE-ATAC libraries from mouse FFPE spleen. Left panel: fragment length distribution (counts); Right panel: fragment length distribution (percentage).

c and **d**: Gene activity projections comparing: (**c**) fresh mouse spleen (scATAC-Seq; see also Supplementary Fig. 4) and (**d**) FFPE mouse spleen (scFFPE-ATAC). Panels shown side-by-side for direct comparison using identical gene sets.
Source data are provided as a Source Data file for Supplementary Fig. 7a-d.

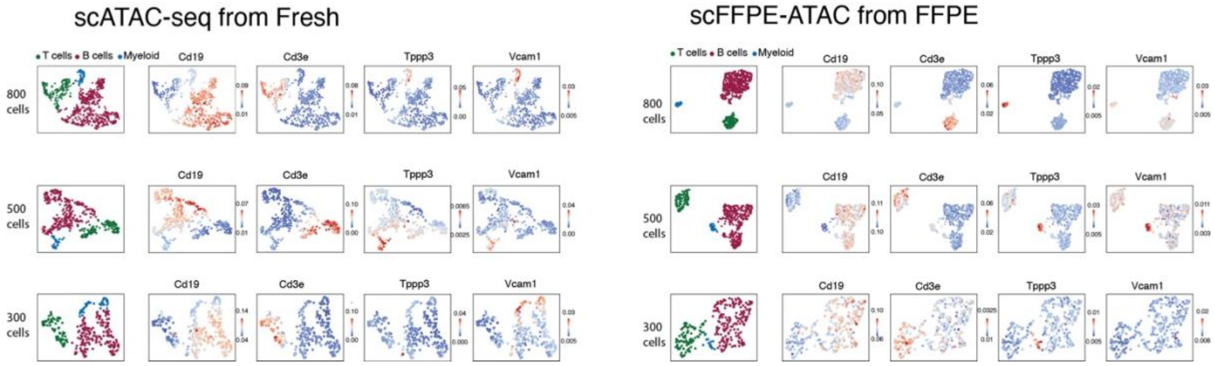

Supplementary Figure 8: Sensitivity comparison between conventional scATAC-Seq in fresh mouse spleen (Left panel) and scFFPE-ATAC in FFPE mouse spleen (Right panel). Source data are provided as a Source Data file for Supplementary Fig. 8.

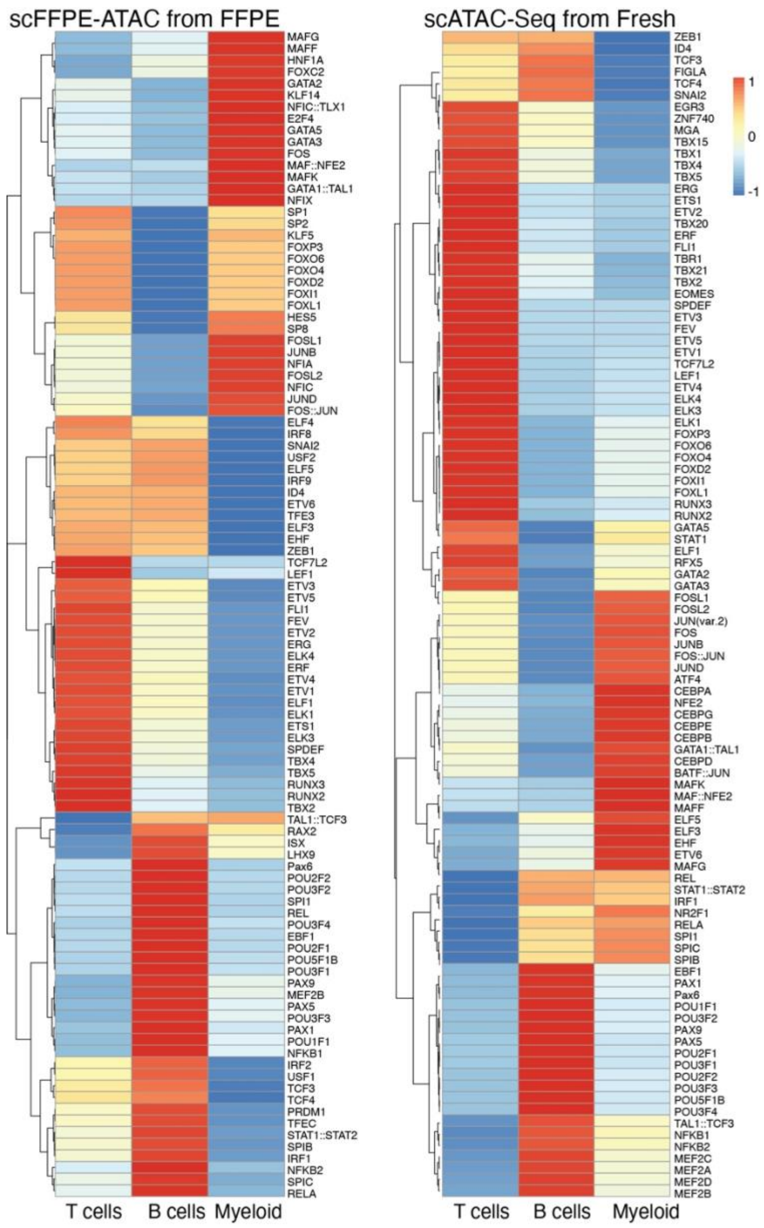

Supplementary Figure 9: **Comparison of transcription factor enrichment based on single-cell chromatin accessibility between scATAC-seq from fresh mouse spleen and scFFPE-ATAC from FFPE mouse spleen.** Source data are provided as a Source Data file for Supplementary Fig. 9.

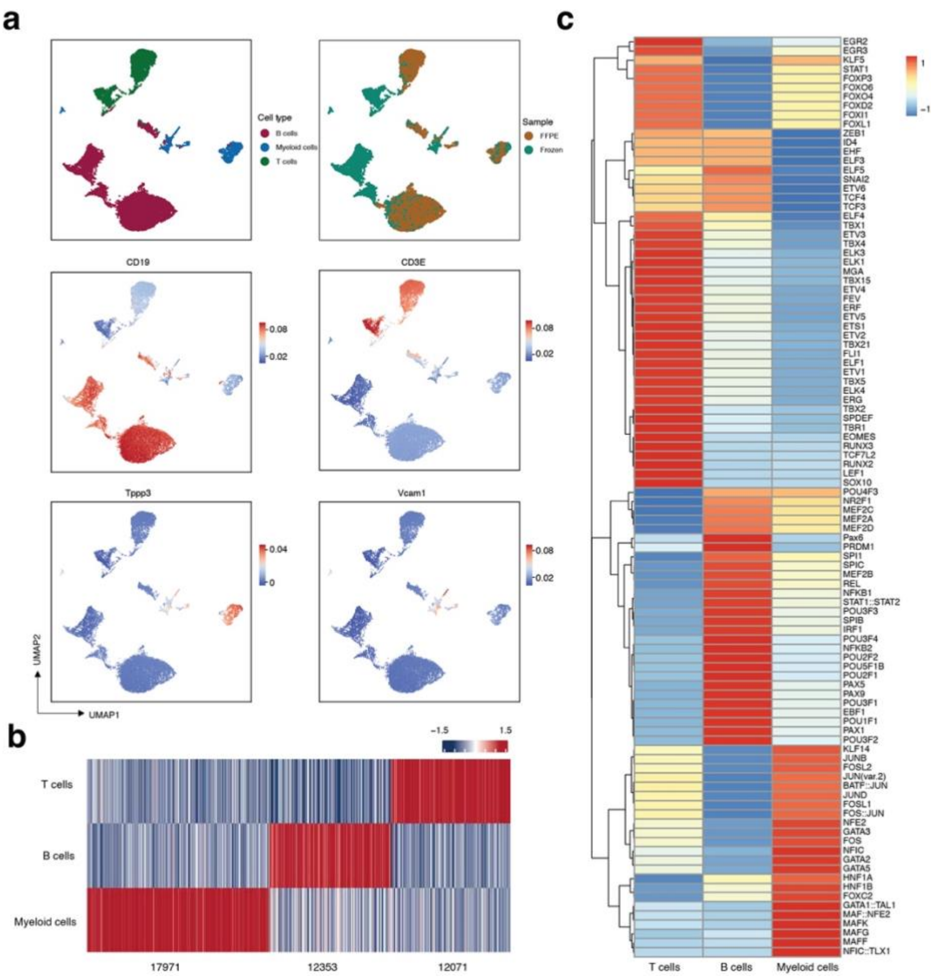

Supplementary Figure 10: **Joint analysis of single-cell chromatin accessibility from fresh and FFPE mouse spleen samples.**

a. Cell type identification using high-dimensional reduction and representative gene activity projection for each cell type.

b. Identified unique accessible chromatin peaks for each cell type.

c. Enriched transcription factors (TFs) associated with unique accessible chromatin peaks for each cell type.

Source data are provided as a Source Data file for Supplementary Fig. 10a-c.

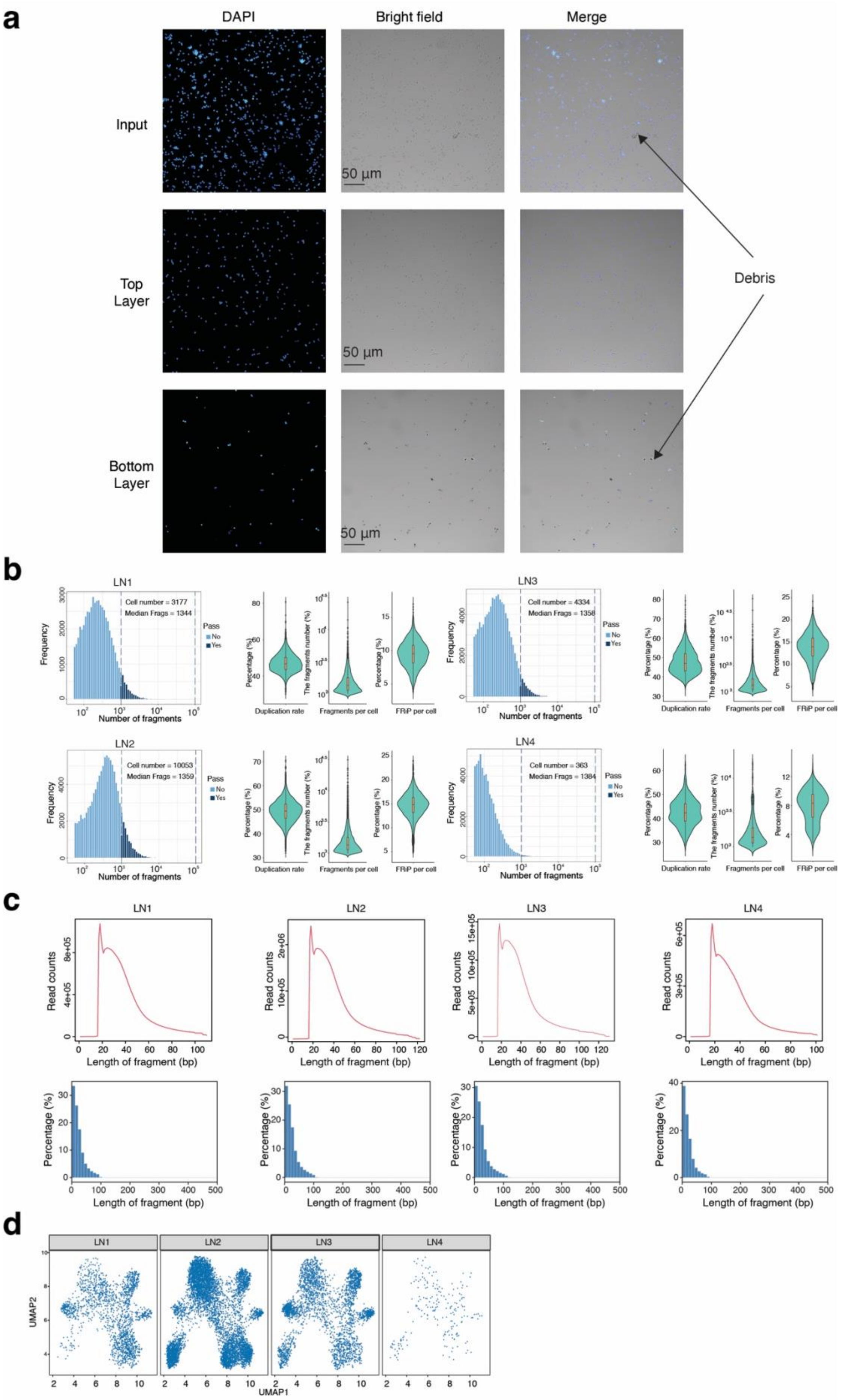
a
DAPI
Bright field
Merge
Input
Top Layer
Bottom Layer
50 µm
Debris
b
LN1
Cell number = 3177
Median Frags = 1344
LN2
Cell number = 10053
Median Frags = 1359
LN3
Cell number = 4334
Median Frags = 1358
LN4
Cell number = 363
Median Frags = 1384
Pass
No
Yes
Frequency
Number of fragments
Percentage (%)
The fragments number (%)
Duplication rate
Fragments per cell
FRiP per cell
c
LN1
LN2
LN3
LN4
Read counts
Length of fragment (bp)
Percentage (%)
d
LN1
LN2
LN3
LN4
UMAP2
UMAP1

Supplementary Figure 11: **scFFPE-ATAC in long-term archived human lymph node FFPE samples.**

a. Representative microscopy images of purified human lymph node FFPE nuclei with density gradient centrifugation from the input, top layer, and bottom layer. Three independent experiments were performed, showing consistent results.

b. Frequency distribution of decoded cell barcodes and quality control metrics for scFFPE-ATAC in human lymph node FFPE samples 1–4 (LN1-4).

Violin plots showing duplication rate, fragments per cell, and FRiP per cell for LN1–4. The box boundaries represent the interquartile range (IQR), spanning from the first quartile (Q1) to the third quartile (Q3).

LN1: Duplication rate (*n* = 3177, max = 80.74, min = 30.22, Q1 = 43.6, median = 46.73, Q3 = 50.27); fragments per cell (*n* = 3177, max = 26,799, min = 1001, Q1 = 1129, median = 1344, Q3 = 1786); FRiP per cell (*n* = 3177, max = 17.04, min = 3.83, Q1 = 8.15, median = 9.56, Q3 = 10.84).

LN2: Duplication rate (*n* = 10,053, max = 70.61, min = 32.00, Q1 = 46.44, median = 49.50, Q3 = 52.31); fragments per cell (*n* = 10,053, max = 31,202, min = 1001, Q1 = 1141, median = 1359, Q3 = 1816); FRiP per cell (*n* = 10,053, max = 24.11, min = 4.78, Q1 = 13.21, median = 14.84, Q3 = 16.33).

LN3: Duplication rate (*n* = 4334, max = 79.34, min = 33.11, Q1 = 43.07, median = 46.94, Q3 = 51.99); fragments per cell (*n* = 4334, max = 59,462, min = 1001, Q1 = 1135, median = 1358, Q3 = 1809); FRiP per cell (*n* = 4334, max = 24.21, min = 4.39, Q1 = 11.91, median = 14.01, Q3 = 15.93).

LN4: Duplication rate (*n* = 363, max = 62.20, min = 28.77, Q1 = 38.83, median = 42.28, Q3 = 46.07); fragments per cell (*n* = 363, max = 16,414, min = 1002, Q1 = 1144.5, median = 1384, Q3 = 1894.5); FRiP per cell (*n* = 363, max = 13.25, min = 2.84, Q1 = 6.36, median = 8.33, Q3 = 9.52).

c. Fragment size distribution of scFFPE-ATAC libraries from human lymph node (LN1-4) FFPE samples. Top panel: fragment length distribution (counts); Bottom panel: fragment length distribution (percentage).

d. Distribution of decoded single cells from scFFPE-ATAC in each human lymph node FFPE sample.

Source data are provided as a Source Data file for Supplementary Fig. 11b-d.

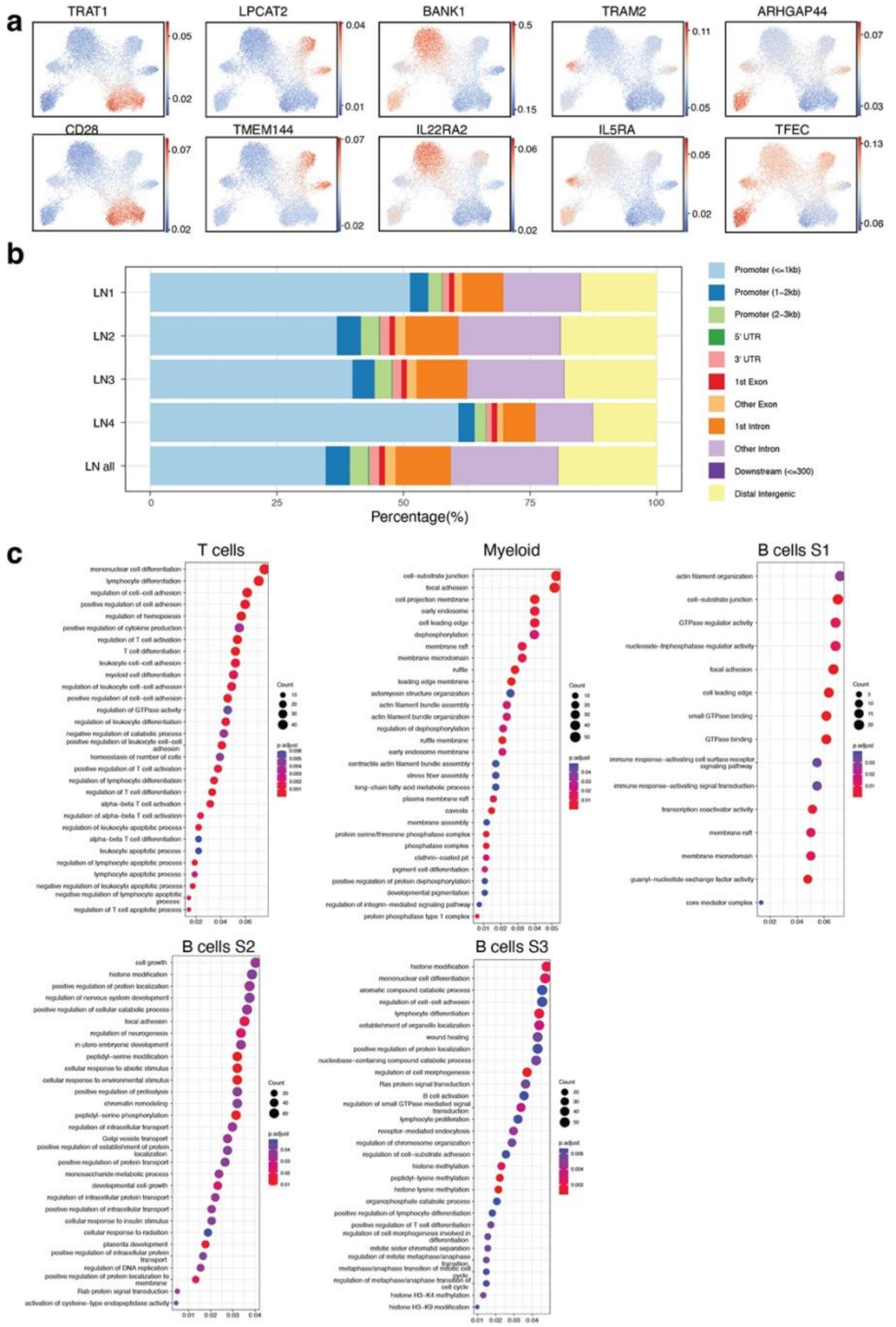

Supplementary Figure 12: **Epigenetic features revealed by scFFPE-ATAC in human lymph node FFPE samples.**

- **a.** Representative gene activity projection from scFFPE-ATAC data.
- **b.** Genomic annotation of accessible chromatin peaks in each lymph node sample.
- **c.** Top enriched biological processes from gene ontology analysis for each cell type identified in the lymph node.

Source data are provided as a Source Data file for Supplementary Fig. 12a-c.

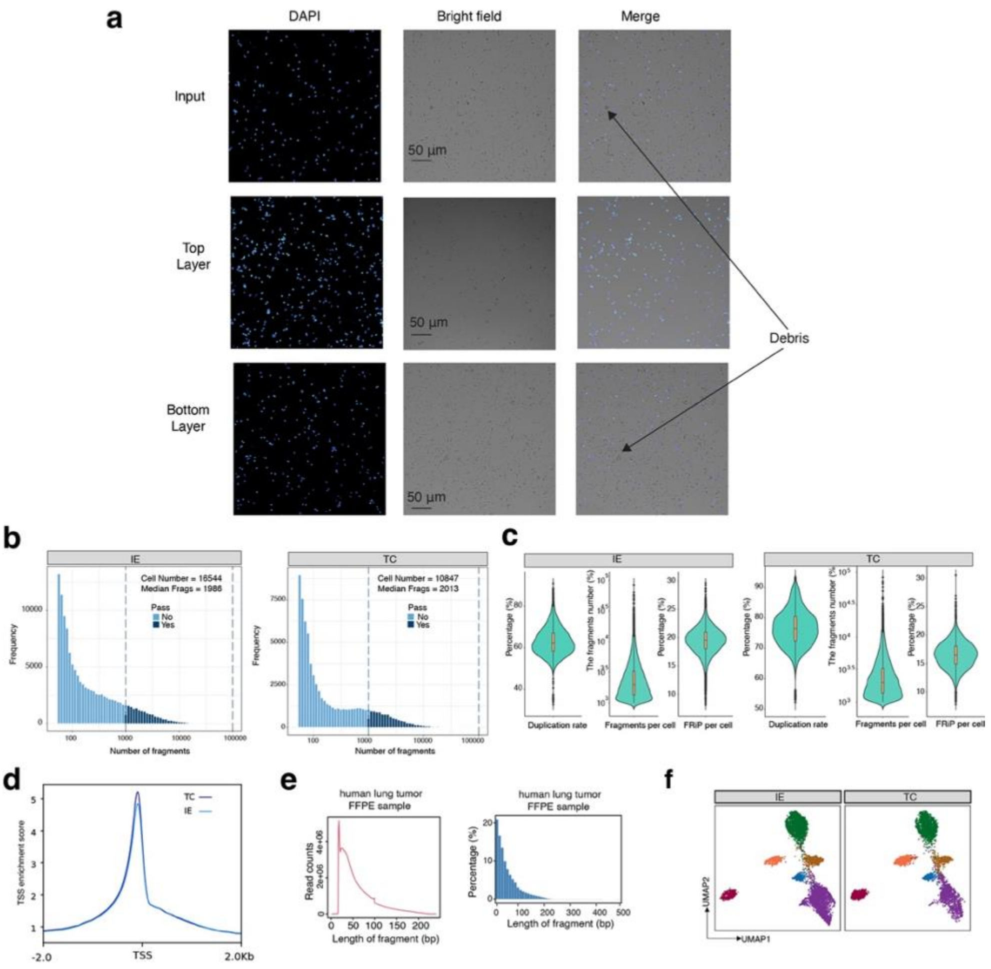

Supplementary Figure 13: **scFFPE-ATAC profiling of the human lung tumor center and invasive edge in human lung cancer FFPE samples.**

a. Representative microscopy images of purified human lung tumor FFPE nuclei with density gradient centrifugation from the input, top layer, and bottom layer. Three independent experiments were performed, showing consistent results.

b. Frequency distribution of detected cell barcodes in the tumor center (TC) and invasive edge (IE) of human lung cancer FFPE samples from scFFPE-ATAC.

c. Duplicate rate, number of unique fragments, and fraction of reads in peaks (FRiP) detected by scFFPE-ATAC in the tumor center (TC) and invasive edge (IE).

The box boundaries represent the interquartile range (IQR), spanning from the first quartile (Q1) to the third quartile (Q3).

IE**:** Duplication rate (n = 17,143, max = 24.21, min = 3.99, lower whisker = 5.24, Q1 = 11.62, median = 14.05, Q3 = 15.88, upper whisker = 22.25); fragments per cell (n = 15,825, max = 63,875, min = 1001, lower whisker = 1001, Q1 = 1370, median = 1995, Q3 = 3286, upper whisker = 6160); FRiP per cell (n = 15,825, max = 29.62, min = 11.13, lower whisker = 14.16, Q1 = 18.27, median = 19.67, Q3 = 21.01, upper whisker = 25.12).

TC: Duplication rate (n = 15,825, max = 90.85, min = 32.69, lower whisker = 46.54, Q1 = 58.52, median = 62.50, Q3 = 67.26, upper whisker = 80.36); fragments per cell (n = 15,825, max = 63,875, min = 1001, lower whisker = 1001, Q1 = 1370, median = 1995, Q3 = 3286, upper whisker = 6160); FRiP per cell (n = 15,825, max = 29.62, min = 11.13, lower whisker = 14.16, Q1 = 18.27, median = 19.67, Q3 = 21.01, upper whisker = 25.12).

d. Transcription start site (TSS) enrichment from scFFPE-ATAC in the tumor center (TC) and invasive edge (IE).

e. Fragment size distribution of scFFPE-ATAC libraries from human lung FFPE samples (merged TC and IE). Left panel: fragment length distribution (counts); Right panel: fragment length distribution (percentage).

f. Distribution of detected cells from scFFPE-ATAC across different clusters in the invasive edge (IE) and tumor center (TC).

Source data are provided as a Source Data file for Supplementary Fig. 13b-f.

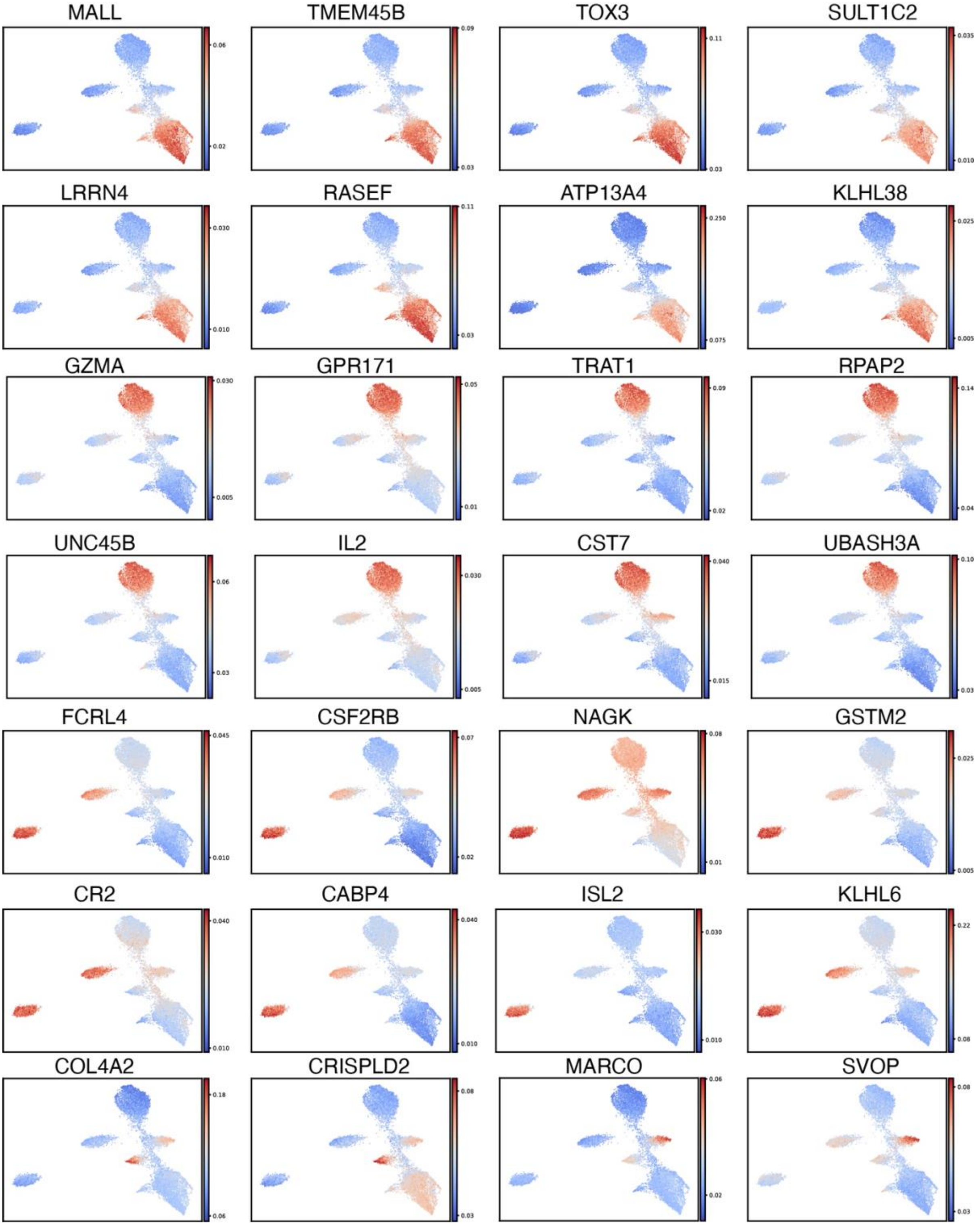

Supplementary Figure 14: **Gene activity projection predicted from scFFPE-ATAC for representative genes in human lung cancer FFPE samples.**

Source data are provided as a Source Data file for Supplementary Fig. 14.

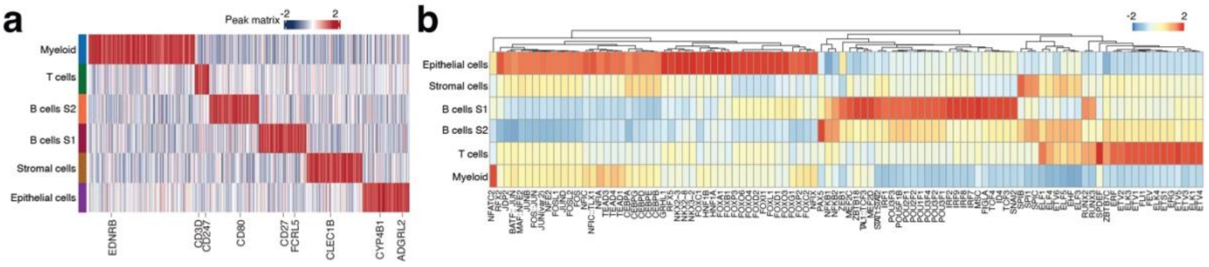

Supplementary Figure 15: **Unique accessible chromatin peaks from scFFPE-ATAC for representative genes (a) and enriched transcriptional factors (TFs) in each cell type from human FFPE lung cancer.**

Source data are provided as a Source Data file for Supplementary Fig. 15a, 15b.

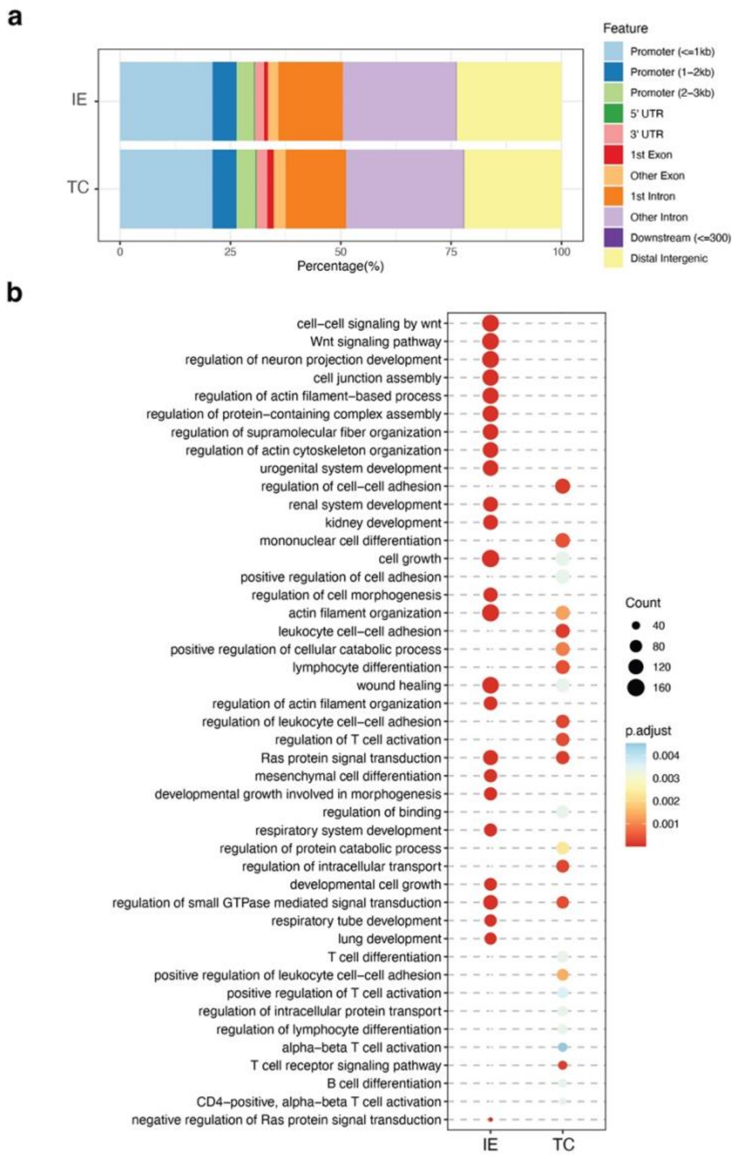

Supplementary Figure 16: **Unique epigenomic features identified from scFFPE-ATAC in epithelial cells from the tumor center and invasive edge of human lung cancer.**

a. Genomic annotation of unique accessible chromatin peaks from epithelial cells in the tumor center (TC) and invasive edge (IE).

b. Biological processes from gene ontology analysis of unique accessible chromatin peaks from epithelial cells in TC and IE.

Source data are provided as a Source Data file for Supplementary Fig. 16a, 16b.

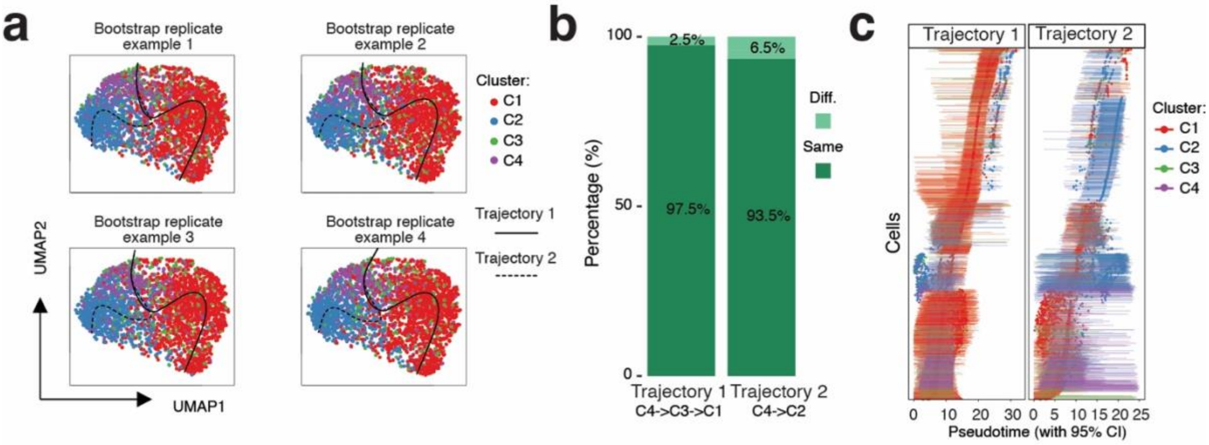

Supplementary Figure 17: Bootstrap validation of trajectories prediction in human lung tumor.

a. Representative trajectories examples inferred from 1000 bootstrap replicates.

b. Branch support proportions calculated across 1,000 bootstrap replicates.

c. Bootstrap-derived pseudotime estimates for Slingshot lineages. Left: Lineage 1; Right: Lineage 2. Each row represents a single cell, ordered by its bootstrap median pseudotime. The horizontal position of each point indicates the median pseudotime estimate, with horizontal error bars representing the 95% confidence interval across 1,000 bootstrap replicates. Cells are colored according to their cluster assignments, illustrating the distribution of clusters along each trajectory.

Source data are provided as a Source Data file for Supplementary Fig. 17a-c.

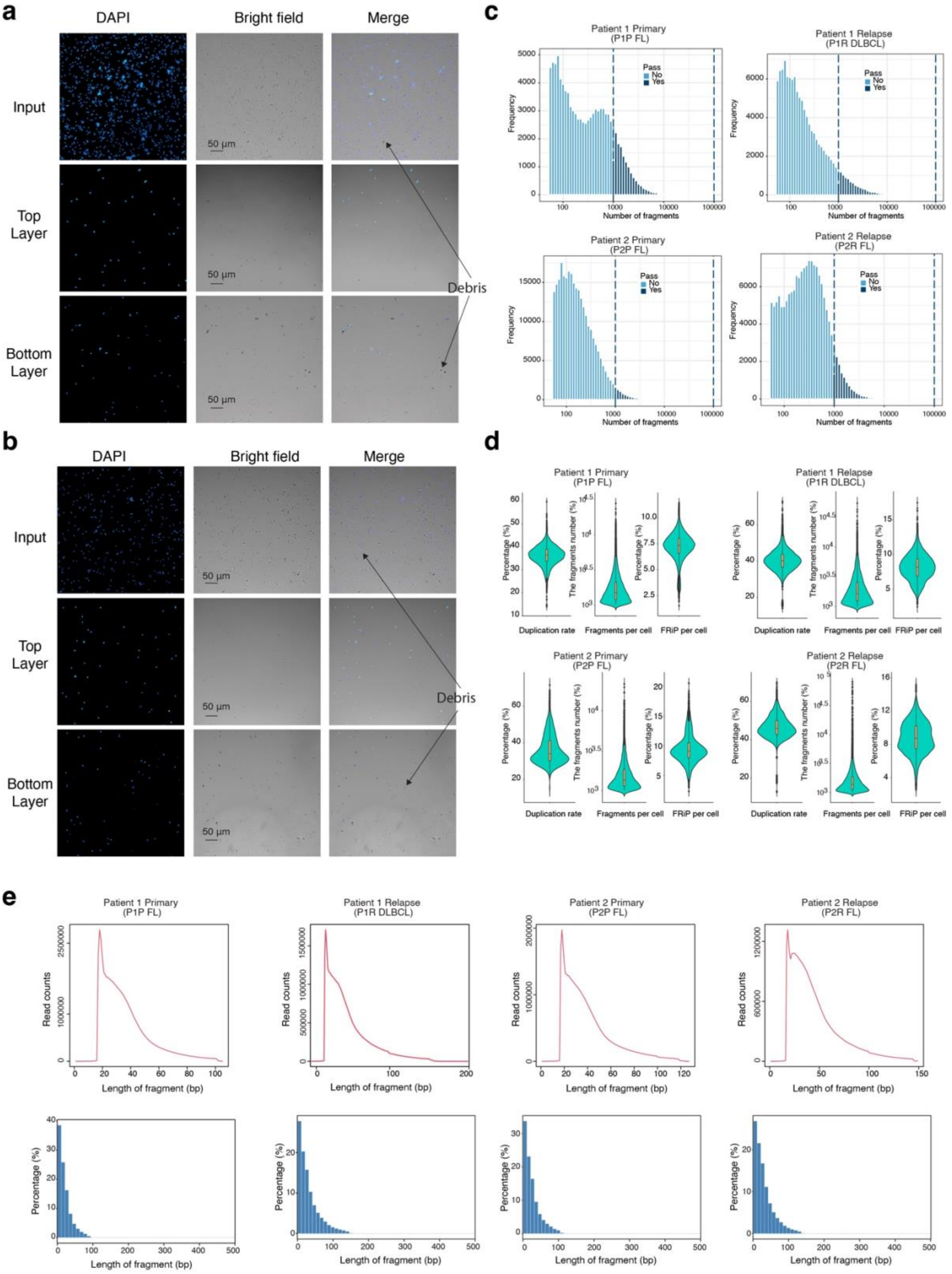

Supplementary Figure 18: **scFFPE-ATAC in clinically archived paired primary and relapse human lymphoma.**

a. Representative microscopy images of purified primary human follicular lymphoma FFPE nuclei with density gradient centrifugation from the input, top layer, and bottom layer. Three independent experiments were performed, showing consistent results.
b. Representative microscopy images of purified relapse human follicular lymphoma FFPE nuclei with density gradient centrifugation from the input, top layer, and bottom layer. Three independent experiments were performed, showing consistent results.
c. The frequency distribution of detected cell barcodes in each sample from scFFPE-ATAC.
d. The duplicate rate, number of unique fragments, and fraction of reads in peaks (FRiP) detected in each sample from scFFPE-ATAC. The box boundaries represent the interquartile range (IQR), spanning from the first quartile (Q1) to the third quartile (Q3).
Patient 1 – Primary: Duplication rate (*n* = 10,999, max = 59.6, min = 13.92, Q1 = 34.4, median = 36.74, Q3 = 39.13); fragments per cell (*n* = 10,999, max = 23,320, min = 1000, Q1 = 1192.5, median = 1491, Q3 = 2077); FRiP per cell (*n* = 10,999, max = 11.45, min = 3.66, Q1 = 6.96, median = 7.56, Q3 = 8.20).
Patient 1 – Relapse: Duplication rate (*n* = 6586, max = 72.22, min = 13.99, Q1 = 37.9, median = 40.98, Q3 = 44.39); fragments per cell (*n* = 6586, max = 54,360, min = 1000, Q1 = 1234, median = 1624, Q3 = 2458); FRiP per cell (*n* = 6586, max = 17.47, min = 4.26, Q1 = 7.75, median = 8.71, Q3 = 9.78).
Patient 2 – Primary: Duplication rate (*n* = 5004, max = 71.54, min = 14.28, Q1 = 30.58, median = 34.69, Q3 = 42.85); fragments per cell (*n* = 5004, max = 16,984, min = 1000, Q1 = 1133, median = 1338, Q3 = 1786.5); FRiP per cell (*n* = 5004, max = 20.43, min = 4.16, Q1 = 8.62, median = 9.58, Q3 = 11.06).
Patient 2 – Relapse: Duplication rate (*n* = 9472, max = 69.87, min = 13.31, Q1 = 43.02, median = 45.88, Q3 = 49.63); fragments per cell (*n* = 9472, max = 81,034, min = 1000, Q1 = 1140, median = 1357, Q3 = 1829); FRiP per cell (*n* = 9472, max = 15.18, min = 4.49, Q1 = 8.10, median = 9.30, Q3 = 10.52).
e. Fragment size distribution of scFFPE-ATAC libraries from paired primary and relapse human FFPE lymphoma (merged patient 1 and patient 2). Top panel: fragment length distribution (counts); Bottom panel: fragment length distribution (percentage).
Source data are provided as a Source Data file for Supplementary Fig. 18c-e.

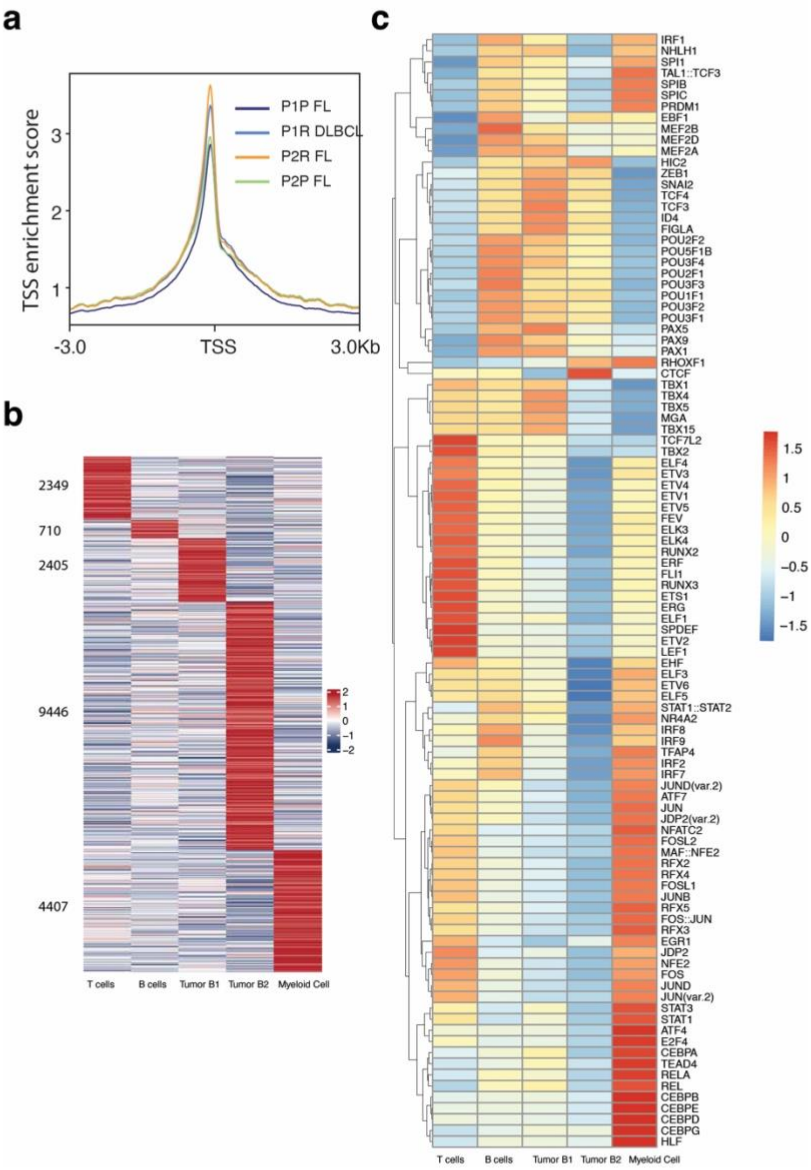

Supplementary Figure 19: **Epigenetic features identified for each cell type in human lymphoma FFPE samples using scFFPE-ATAC.**

a. Transcription start site (TSS) enrichment in each human lymphoma sample.

b. Unique accessible chromatin peaks for each cell type, with the number of peaks labeled on the left side.

c. Enriched transcription factors on unique accessible chromatin peaks for each cell type.
Source data are provided as a Source Data file for Supplementary Fig. 19a-c.

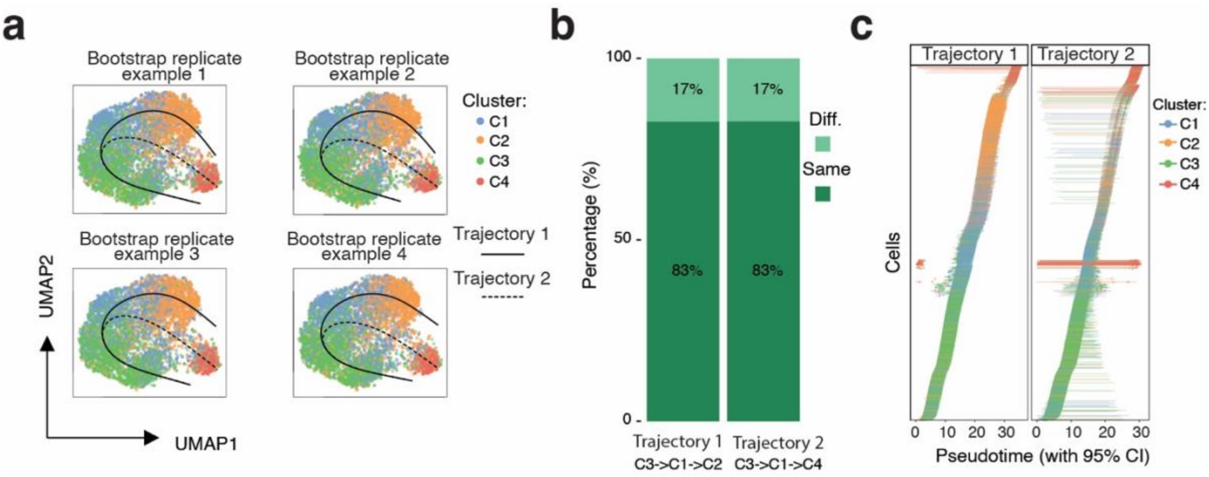

Supplementary Figure 20: Bootstrap validation of trajectories prediction in primary and relapse human lymphoma.

a. Representative trajectories examples inferred from 1000 bootstrap replicates.
b. Branch support proportions calculated across 1,000 bootstrap replicates.
c. Bootstrap-derived pseudotime estimates for Slingshot lineages. Left: Lineage 1; Right: Lineage 2. Each row represents a single cell, ordered by its bootstrap median pseudotime. The horizontal position of each point indicates the median pseudotime estimate, with horizontal error bars representing the 95% confidence interval across 1,000 bootstrap replicates. Cells are colored according to their cluster assignments, illustrating the distribution of clusters along each trajectory.

Source data are provided as a Source Data file for Supplementary Fig. 20a-c.

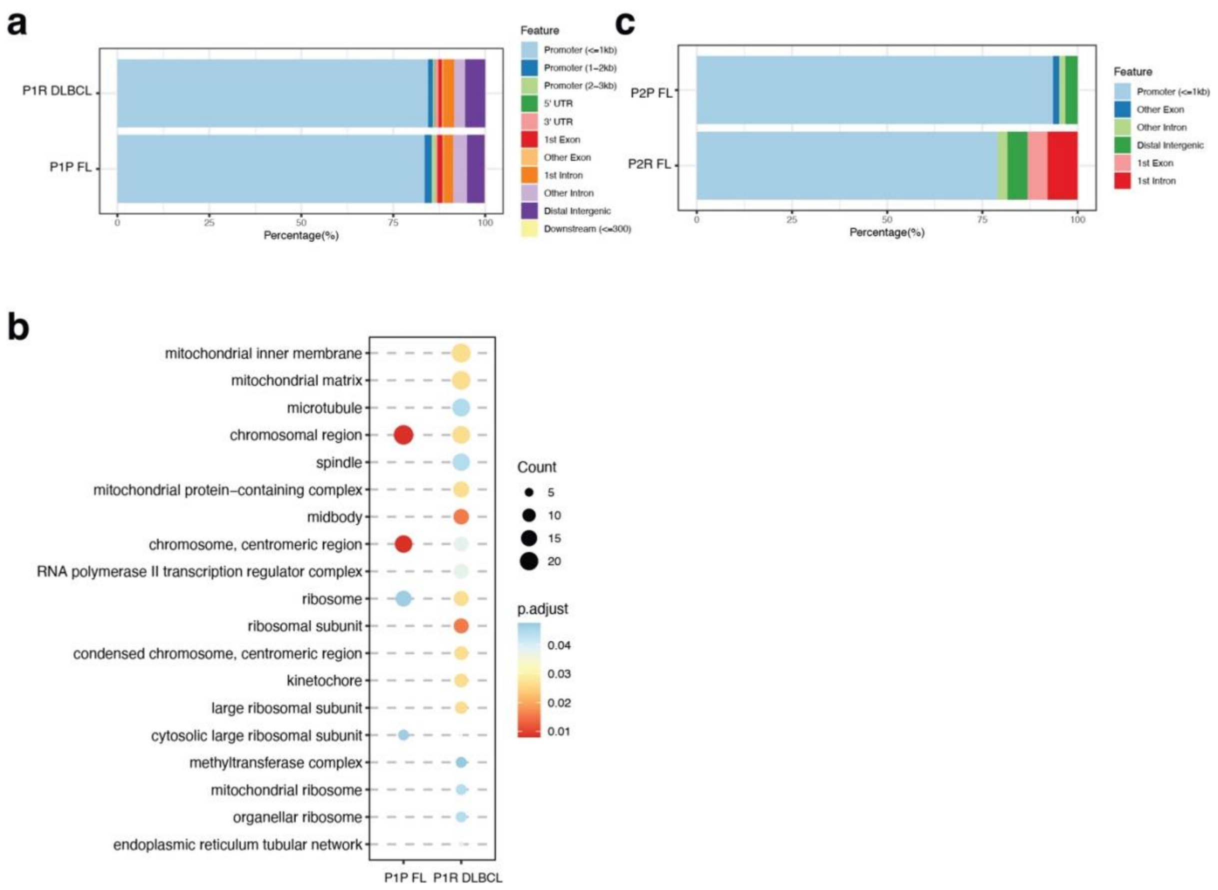

Supplementary Figure 21: **Epigenomic features identified from scFFPE-ATAC in paired primary and relapse human lymphoma.**

a. Genomic annotation of differential accessible chromatin peaks in cell type tumor B1 between patient 1 primary follicular lymphoma (P1P FL) and patient 1 relapse diffuse large B cell lymphoma (P1R DLBCL).

b. Biological process pathway enrichment from gene ontology analysis of differential accessible chromatin peaks in cell type tumor B1 between P1P FL and P1R DLBCL.

c. Genomic annotation of differential accessible chromatin peaks in cell type tumor B2 between patient 2 primary follicular lymphoma (P2P FL) and patient 2 relapse follicular lymphoma (P2R FL).

Source data are provided as a Source Data file for Supplementary Fig. 21a-c.

Supplementary Technical Note 1 & 2:

Supplementary Technical Note 1: Nuclei Isolation Optimization and Quality Control
The quality and purity of isolated nuclei are critical determinants of the success and reproducibility of scFFPE-ATAC sequencing libraries. This conclusion is based on experiments in which we performed scFFPE-ATAC on mouse FFPE spleen with and without purification of nuclei by density gradient centrifugation.

To obtain intact nuclei, rather than fragmented nuclei with chromatin leakage, the tissue section thickness should be greater than 10 µm. In our protocol development, we identified two major factors influencing nuclei quality and purity: enzymatic digestion efficiency and debris removal. Below, we outline both the experimental considerations and the quality control measures used to optimize nuclei isolation.

1. Enzymatic digestion to release nuclei
Complete enzymatic digestion is essential to release nuclei from the surrounding extracellular matrix. Incomplete digestion can result in nuclear doublets and hinder accurate single-cell chromatin profiling—particularly when distinct cell types, such as T cells and tumor cells, are embedded within fibrotic tissue regions. We optimized the enzymatic digestion step using collagenase and hyaluronidase, noting that both enzyme concentration and digestion time vary significantly by tissue type. To determine optimal conditions, we performed a matrix of digestion experiments varying enzyme concentrations and incubation durations. At each time point, small aliquots were taken and assessed under a fluorescence microscope to evaluate digestion efficiency using nuclear staining (e.g., DAPI or Hoechst) (Supplementary Technical Note Figure 1).

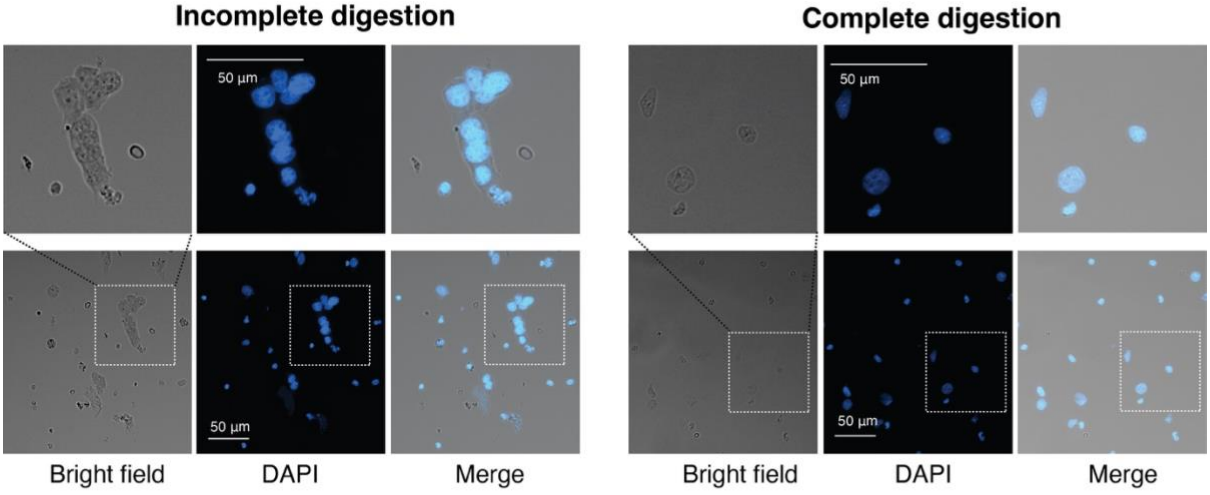

Supplementary Technical Note Figure 1: Comparison of human lung tumor nuclei after complete and incomplete digestion, visualized by nuclear staining under microscopy.

2. Density gradient centrifugation for debris removal
The second key parameter was density gradient centrifugation, which proved essential for removing extracellular matrix components and cellular debris. Without density gradient centrifugation, we observed considerable debris, whereas with density gradient centrifugation, there was little to none (Supplementary Technical Note Figure 2).

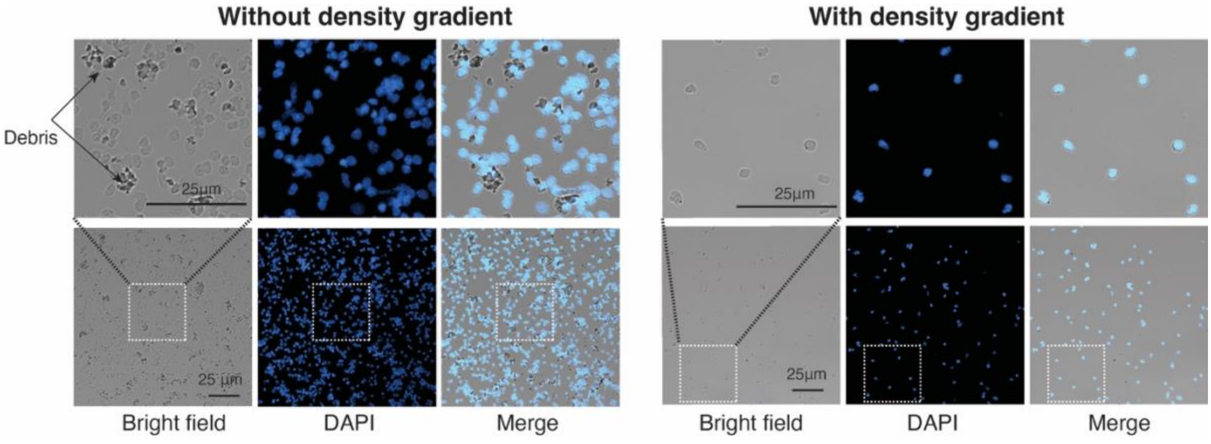

Supplementary Technical Note Figure 2: Comparison of mouse FFPE spleen nuclei with and without density gradient centrifugation. The white box indicates the zoomed-in area of the upper row shown in the lower row.

We evaluated nuclei quality using the following two criteria:

(1). Morphological integrity

Nuclei were examined under a fluorescence microscope following nuclear staining. High-quality nuclei appeared intact, round, and evenly stained, with minimal clumping or fragmentation. The presence of nuclear doublets, irregular shapes, or signs of lysis was used as an indicator of suboptimal enzymatic digestion or incomplete removal of the extracellular matrix.

(2). Extracellular matrix components and cellular debris content

Debris was visually assessed in the stained nuclei preparation together with bright-field microscopy (Supplementary Technical Note Figure 2). Low-quality samples displayed abundant granular debris and extracellular contaminants, which could interfere with downstream enzymatic steps such as Tn5 tagmentation and ligation during the split-and-pool barcoding workflow. We aimed to minimize debris as much as possible, either through multiple rounds of density gradient centrifugation or by incorporating flow cytometric sorting, combining nuclear staining with gating strategies to exclude debris.

We did not observe a major difference in DNA size distribution between the conditions with and without density gradient centrifugation in mouse FFPE spleen nuclei (Supplementary Technical Note Figure 3a). We used cutoffs of 200 bp, 300 bp, 400 bp, and 500 bp to measure the ratio of DNA below and above each cutoff, and the ratios were very similar between the conditions with and without density gradient centrifugation.

We compared single-cell FFPE-ATAC libraries prepared with and without density gradient centrifugation using mouse FFPE spleen tissue. In the scFFPE-ATAC library analysis of nuclei with and without density gradient centrifugation (Supplementary Technical Note Figure 3b–f), we observed lower TSS enrichment, higher duplication rates, and a lower fraction of reads in peaks (FRiP) in the condition without density gradient centrifugation (Supplementary Technical Note Figure 3b, 3c). We were only able to decipher single-cell chromatin accessibility profiles in the condition with density gradient centrifugation, but not

without it, as we could not clearly separate cell types or detect gene activities (Supplementary Technical Note Figure 3d–f). We hypothesize that unpurified nuclei, particularly debris, may promote nonspecific Tn5 binding during tagmentation, leading to low TSS enrichment scores, high duplication rates, and reduced FRiP values compared with gradient-purified nuclei. In addition, debris may reduce ligation efficiency by sequestering DNA oligos, further compromising library quality.

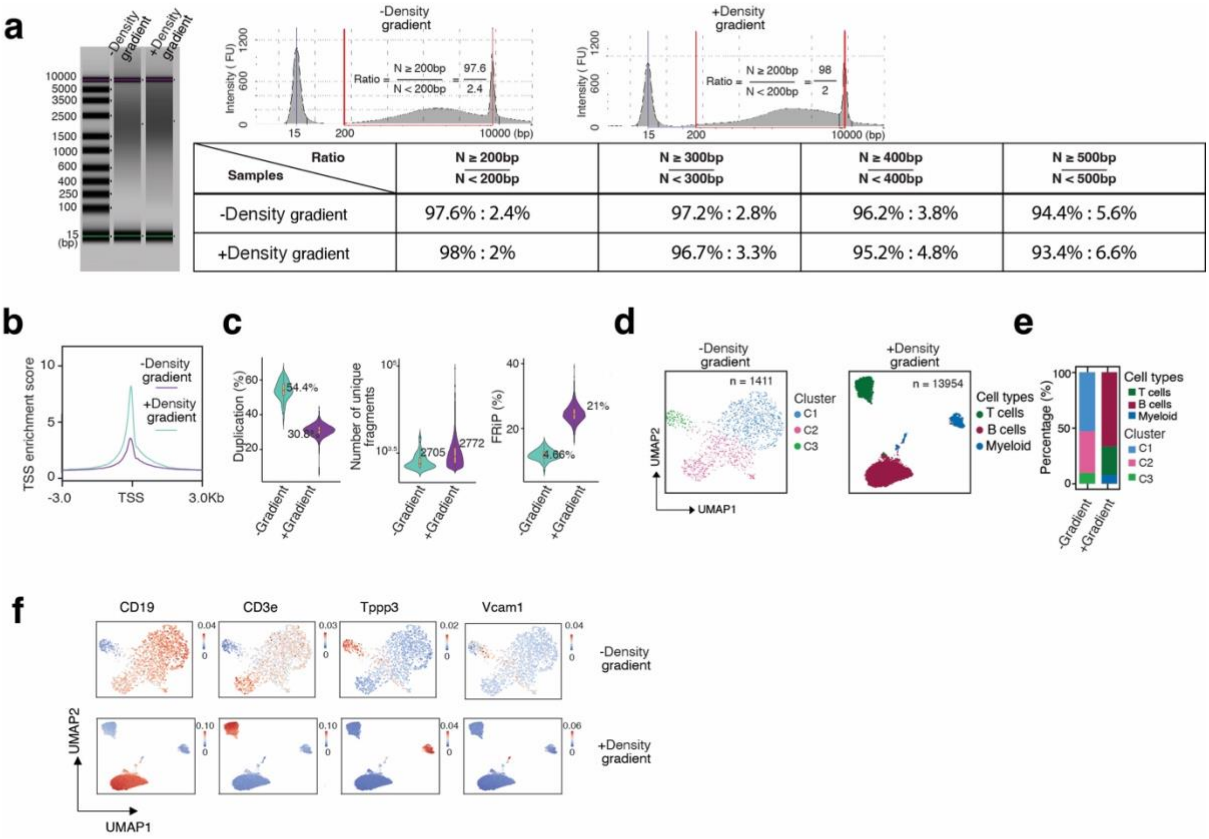

Supplementary Technical Note Figure 3: Comparison of DNA length distribution and scFFPE-ATAC profiles from mouse FFPE spleen nuclei with and without density gradient centrifugation.

- **a.** DNA length distribution. Three independent experiments were performed, showing consistent results.
- **b.** Sequencing signal enrichment at transcription start sites (TSSs).
- **c.** Left: duplication rate; middle: number of unique fragments; right: fraction of reads in peaks. Median values are labeled in each comparison.
- **d.** Identification of cell types from scFFPE-ATAC under different conditions.
- **e.** Proportional distribution of different cell types or clusters.
- **f.** Examples of active genes in each cell type or cluster.

We also purified DNA from human FFPE lymph nodes (stored for 8–12 years), human FFPE lung tumors (stored for 2 years), and paired primary and relapse human FFPE lymphoma samples (stored for 6–13 years) and evaluated the DNA size distribution from these samples

(Supplementary Technical Note Figure 4). The DNA from these samples showed fragmented sizes ranging from ~15 bp to 10,000 bp, spanning very short to long fragments after many years of storage under different conditions. We observed very few DNA fragments shorter than 100 bp across all samples. In all cases, more than 80% of DNA fragments were larger than 200 bp.

We then used cutoffs of 200 bp, 300 bp, 400 bp, and 500 bp to calculate the ratio of DNA fragments below and above each cutoff. These ratios showed diverse distributions among samples (Supplementary Technical Note Figure 4b, 4c). However, despite this diversity in DNA fragment size distributions, we did not observe significantly lower scFFPE-ATAC quality in these samples.

Thus, our results indicate that assessing nuclei purity after nuclei isolation, rather than relying on DNA fragment size distribution, is a more reliable parameter for evaluating suitability prior to scFFPE-ATAC.

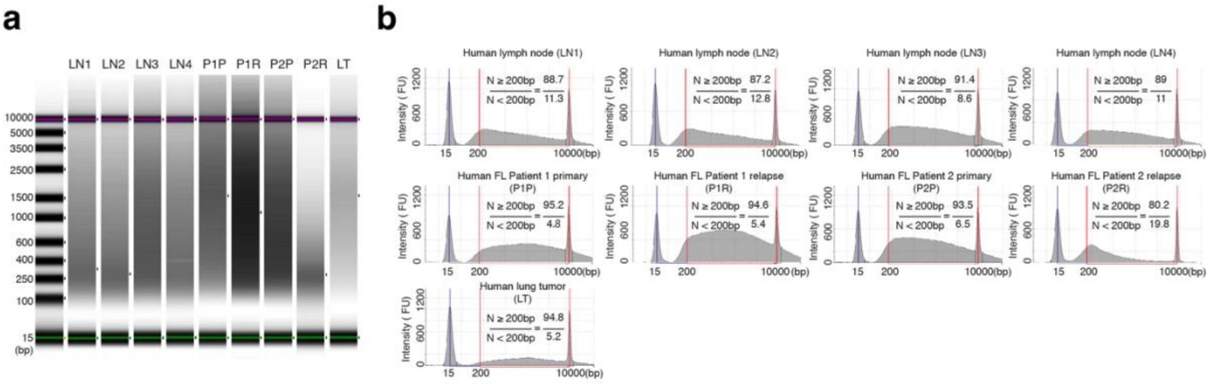

c

Samples \ Ratio	N ≥ 200bp / N < 200bp	N ≥ 300bp / N < 300bp	N ≥ 400bp / N < 400bp	N ≥ 500bp / N < 500bp
Human lymph node (LN1)	88.7% : 11.3%	79% : 21%	70.1% : 29.9%	62.1% : 37.9%
Human lymph node (LN2)	87.2% : 12.8%	76.9% : 23.1%	67.7% : 32.3%	59.7% : 40.3%
Human lymph node (LN3)	91.4% : 8.6%	83.7% : 16.3%	76.0% : 24.0%	68.9% : 31.1%
Human lymph node (LN4)	89.0% : 11.0%	80.4% : 19.6%	72.6% : 27.4%	65.2% : 34.8%
Human FL Patient 1 primary (P1P)	95.2% : 4.8%	89.7% : 10.3%	83.7% : 16.3%	77.8% : 22.2%
Human FL Patient 1 relapse (P1R)	94.6% : 5.4%	88.3% : 11.7%	81.8% : 18.2%	75.4% : 24.6%
Human FL Patient 2 primary (P2P)	93.5% : 6.5%	85.9% : 14.1%	78.6% : 21.4%	71.4% : 28.6%
Human FL Patient 2 relapse (P2R)	80.2% : 19.8%	63.8% : 36.2%	49.9% : 50.1%	39.5% : 60.5%
Human lung tumor (LT)	94.8% : 5.2%	90.4% : 9.6%	85.2% : 14.8%	79.9% : 20.1%

Supplementary Technical Note Figure 4: Quantification of DNA length distribution in archived human FFPE samples, including lymph nodes (LN1–4), Patient 1 primary follicular lymphoma (P1P), Patient 1 relapse follicular lymphoma (P1R), Patient 2 primary follicular lymphoma (P2P), Patient 2 relapse follicular lymphoma (P2R), and a human lung tumor (LT).

- **a.** Gel images from Tapestation. Three independent experiments were performed, showing consistent results.
- **b.** DNA length distribution ratio of fragments ≥200 bp vs. <200 bp.
- **c.** DNA length distribution ratios with cutoffs at 200 bp, 300 bp, 400 bp, and 500 bp.

Supplementary Technical Note 2: Tn5 transposase reaction system in the scFFPE-ATAC.

The final concentration of Tn5 transposase in our study was determined following a previous report[1]. A brief description of the process is as follows: Homemade Tn5 was diluted with dilution buffer (50 mM Tris, 100 mM NaCl, 0.1 mM EDTA, 1 mM DTT, 0.1% NP-40, and 50% glycerol) to different concentrations. Tagmentation was then performed on 50 ng of purified genomic DNA instead of cells. We quantified the number of cycles required to reach one-third of the plateau fluorescence by qPCR and determined the final dilution factor of homemade Tn5 that most closely matched the number of cycles observed with Nextera TDE1.

To avoid potential nuclei aggregation during Tn5 tagmentation, we used a 100 μL reaction system with 5 μL of Tn5 transposase. The final concentration of Tn5 transposase, however, remained unchanged. We hypothesized that the concentration of Tn5 transposase, rather than the reaction volume, primarily affects tagmentation efficiency.

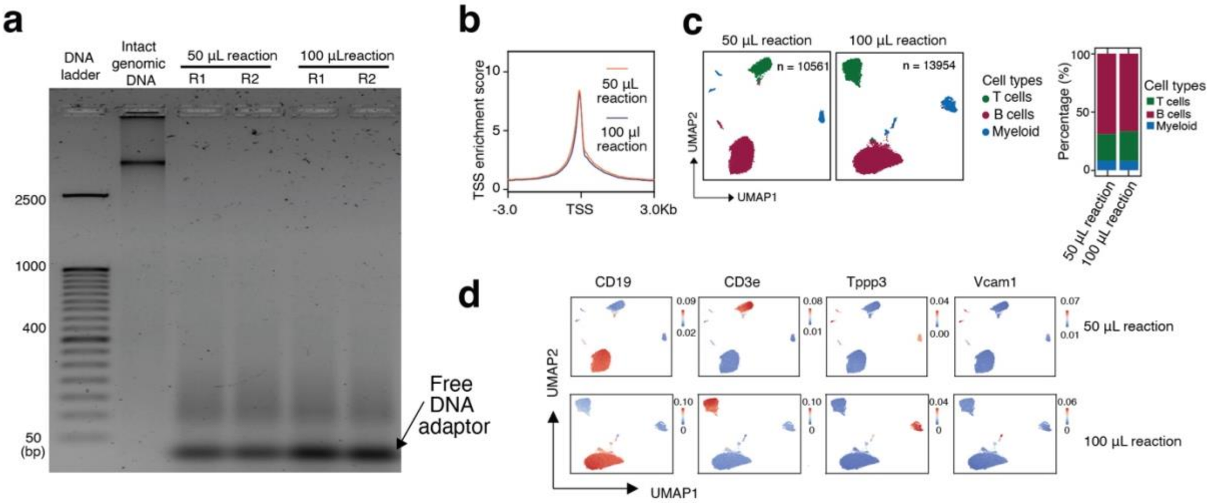

a. **Supplementary Technical Note Figure 5: Different reactions with the same concentration of Tn5 transposase for scFFPE-ATAC.**
a, *In vitro* Tn5 transposase activity comparison using 50 μL and 100 μL reaction volumes with the same concentration. Two replicates were included for each assay. R1 = replicate 1; R2 = replicate 2. Three independent experiments were performed, showing consistent results. The entire gel is shown without cropping.

b, The TSS enrichment score in single-cell FFPE-ATAC with different reaction volumes.
c, Cell type identification in single-cell FFPE-ATAC with different reaction volumes (left panel: UMAP; right panel: proportion comparison).
d, Examples of active genes in each cell type.

To confirm our hypothesis that the concentration of Tn5 transposase does not have a dosage effect, we performed two experiments:

1. We tested Tn5 transposase activity in both 50 μL (with 2.5 μL Tn5 transposase) and 100 μL (with 5 μL Tn5) reaction volumes, keeping the final concentration of Tn5

transposase constant by using human genomic DNA. We observed that the activity was similar in both reaction volumes, with comparable size distributions of genomic DNA after tagmentation (Supplementary Technical Note Figure 5a). (Note: The absolute number of free DNA adapters is higher in the 100 μL reaction volume because twice the amount of Tn5 was added, but this did not affect the efficiency of the tagmentation reaction.)

2. We conducted a scFFPE-ATAC experiment using mouse FFPE spleen in a 50 μL reaction system and found that the quality of scFFPE-ATAC was comparable to that obtained with the 100 μL system in terms of TSS enrichment, cell type detection, and single-cell epigenetic profiles, among other metrics (Supplementary Technical Note Figure 5b–d).

In summary, our results indicate that the concentration of Tn5 transposase, rather than the reaction volume or total dosage, is the key factor affecting tagmentation efficiency. Therefore, our results indicate that both the 50 μL and 100 μL reaction systems are effective.

Supplementary Tables 1-24:

Supplementary tables 1: Details of the experiments and sample information.

Supplementary tables 2: **Summary of DNA oligo sequences used in this study.**

Supplementary Table 3: **Cell type specific active genes predicted from scFFPE-ATAC in mouse FFPE spleen.** A two-sided Wilcoxon rank-sum test was applied in the significance test. The false discovery rate (FDR) was adjusted using the Benjamini–Hochberg (BH) method.

Supplementary Table 4: **Cell type specific active genes predicted from single cell-ATAC in mouse fresh spleen**. A two-sided Wilcoxon rank-sum test was applied in the significance test. The false discovery rate (FDR) was adjusted using the Benjamini–Hochberg (BH) method.

Supplementary Table 5: **Cell type specific accessible chromatin peaks from scFFPE-ATAC identified in mouse FFPE spleen.** A two-sided t-test was applied in the significance test. The false discovery rate (FDR) was adjusted using the Benjamini–Hochberg (BH) method.

Supplementary Table 6: **Cell type specific accessible chromatin peaks from single cell-ATAC identified in mouse fresh spleen.** A two-sided t-test was applied in the significance test. The false discovery rate (FDR) was adjusted using the Benjamini–Hochberg (BH) method.

Supplementary Table 7: **Cell type specific active genes predicted from joined single cell-ATAC in mouse fresh spleen and scFFPE-ATAC in mouse FFPE spleen.** A two-sided t-test was applied in the significance test. The false discovery rate (FDR) was adjusted using the Benjamini–Hochberg (BH) method.

Supplementary Table 8: **Cell type specific accessible chromatin peaks from joined single cell-ATAC in mouse fresh spleen and scFFPE-ATAC in mouse FFPE spleen.** A two-sided t-test was applied in the significance test. The false discovery rate (FDR) was adjusted using the Benjamini–Hochberg (BH) method.

Supplementary Table 9: Cell type specific active genes predicted from scFFPE-ATAC in human FFPE lymph node. A two-sided Wilcoxon rank-sum test was applied in the significance test. The false discovery rate (FDR) was adjusted using the Benjamini–Hochberg (BH) method.

Supplementary Table 10: Cell type specific accessible chromatin peaks from scFFPE-ATAC in human FFPE lymph node. A two-sided t-test was applied in the significance test. The false discovery rate (FDR) was adjusted using the Benjamini–Hochberg (BH) method.

Supplementary Table 11: List of gene ontology enriched from cell type specific accessible chromatin peaks from scFFPE-ATAC human FFPE lymph node. A one-sided Fisher's exact test was applied in the significance test. The adjusted p-values were corrected using the Benjamini–Hochberg (BH) method.

Supplementary Table 12: Cell type specific active genes predicted from scFFPE-ATAC in tumor center (TC) and invasive edge (IE) of human FFPE lung cancer. A two-sided Wilcoxon rank-sum test was applied in the significance test. The false discovery rate (FDR) was adjusted using the Benjamini–Hochberg (BH) method.

Supplementary Table 13: Cell type specific accessible chromatin peaks from scFFPE-ATAC in tumor center (TC) and invasive edge (IE) of human FFPE lung cancer. A two-sided t-test was applied in the significance test. The false discovery rate (FDR) was adjusted using the Benjamini–Hochberg (BH) method.

Supplementary Table 14: Differential accessible chromatin peaks from the epithelial cells in tumor center (TC) and invasive edge (IE) of human FFPE lung cancer. A two-sided t-test was applied in the significance test. The false discovery rate (FDR) was adjusted using the Benjamini–Hochberg (BH) method.

Supplementary Table 15: List of gene ontology enriched from the epithelial cells in tumor center (TC) and invasive edge (IE) of human FFPE lung cancer. A one-sided Fisher's exact test was applied in the significance test. The adjusted p-values were corrected using the Benjamini–Hochberg (BH) method.

Supplementary Table 16: Cluster-specific accessible chromatin peaks identified in pseudotime trajectory analysis. A two-sided Wilcoxon rank-sum test was applied in the significance test. The false discovery rate (FDR) was adjusted using the Benjamini–Hochberg (BH) method.

Supplementary Table 17: Cluster-specific transcription factor enrichment identified in pseudotime trajectory analysis.

Supplementary Table 18: Cell type specific active genes predicted from scFFPE-ATAC in paired primary and relapse human lympoma. A two-sided Wilcoxon rank-sum test was applied in the significance test. The false discovery rate (FDR) was adjusted using the Benjamini–Hochberg (BH) method.

Supplementary Table 19: Cell type specific accessible chromatin peaks from scFFPE-ATAC ATAC in paired primary and relapse human lympoma. A two-sided t-test was applied in the significance test. The false discovery rate (FDR) was adjusted using the Benjamini–Hochberg (BH) method.

Supplementary Table 20: Cluster-specific active genes identified through pseudotime trajectory analysis in paired primary and relapse human lymphoma samples. A two-sided Wilcoxon rank-sum test was applied in the significance test. The false discovery rate

(FDR) was adjusted using the Benjamini–Hochberg (BH) method.

Supplementary Table 21: Differential accessible chromatin peaks from the cell type of Tumor B1 in Patient 1 primary and relapse comparison. A two-sided t-test was applied in the significance test. The false discovery rate (FDR) was adjusted using the Benjamini–Hochberg (BH) method.

Supplementary Table 22: List of gene ontology enriched from differential accessible chromatin peaks from the cell type of Tumor B1 in Patient 1 primary and relapse comparison. A one-sided Fisher's exact test was applied in the significance test. The adjusted p-values were corrected using the Benjamini–Hochberg (BH) method.

Supplementary Table 23: Differential accessible chromatin peaks from the cell type of Tumor B2 in Patient 2 primary and relapse comparison. A two-sided t-test was applied in the significance test. The false discovery rate (FDR) was adjusted using the Benjamini–Hochberg (BH) method.

Supplementary Table 24: Cost estimation per experiment.

References:

1. Ma, S. et al. Chromatin Potential Identified by Shared Single-Cell Profiling of RNA and Chromatin. *Cell* **183**, 1103-1116 e1120 (2020).
